# An Improved WiFi Positioning Method Based on Fingerprint Clustering and Signal Weighted Euclidean Distance

**DOI:** 10.3390/s19102300

**Published:** 2019-05-18

**Authors:** Boyuan Wang, Xuelin Liu, Baoguo Yu, Ruicai Jia, Xingli Gan

**Affiliations:** 1 College of Information and Communication Engineering, Harbin Engineering University, Harbin 150001, China; boyuan@hrbeu.edu.cn; 2 State Key Laboratory of Satellite Navigation System and Equipment Technology, Shijiazhuang 050081, China; yubg@sina.cn (B.Y.); jiaruicai@126.com (R.J.); ganxingli@126.com (X.G.); 3 The 54th Research Institute of China Electronics Technology Group Corporation, Shijiazhuang 050081, China

**Keywords:** WiFi positioning, fingerprint clustering, weighted Euclidean distance, physical distance, weighted K-nearest neighbor

## Abstract

WiFi fingerprint positioning has been widely used in the indoor positioning field. The weighed K-nearest neighbor (WKNN) algorithm is one of the most widely used deterministic algorithms. The traditional WKNN algorithm uses Euclidean distance or Manhattan distance between the received signal strengths (RSS) as the distance measure to judge the physical distance between points. However, the relationship between the RSS and the physical distance is nonlinear, using the traditional Euclidean distance or Manhattan distance to measure the physical distance will lead to errors in positioning. In addition, the traditional RSS-based clustering algorithm only takes the signal distance between the RSS as the clustering criterion without considering the position distribution of reference points (RPs). Therefore, to improve the positioning accuracy, we propose an improved WiFi positioning method based on fingerprint clustering and signal weighted Euclidean distance (SWED). The proposed algorithm is tested by experiments conducted in two experimental fields. The results indicate that compared with the traditional methods, the proposed position label-assisted (PL-assisted) clustering result can reflect the position distribution of RPs and the proposed SWED-based WKNN (SWED-WKNN) algorithm can significantly improve the positioning accuracy.

## 1. Introduction

In indoor environments, global navigation satellite systems (GNSS) can be affected by unfavorable factors, such as signal blocking and multipath propagation, which make it unable to achieve a satisfactory positioning performance [1]. According to the report of the US Environmental Protection Agency, people spend nearly 70–90% of their time indoors [2]. Therefore, it is important to establish an accurate, reliable, and real-time indoor positioning system to satisfy the public demand for indoor positioning. With the rapid development of intelligent terminals, smartphones have more sensors and have become excellent tools for indoor positioning. Additionally, WiFi signal receiving modules have been widely embedded in smartphones, and hotspots have also been covered in many public places, such as office buildings, airports, and shopping malls. Therefore, WiFi fingerprint positioning has become one of the most popular indoor positioning schemes and has been widely used in recent years [3]. The WiFi fingerprint positioning can be divided into the offline stage and the online stage. In the offline stage, the user collects the received signal strength (RSS) from the wireless access points (APs) on each reference point (RP) whose position is known, the coordinates and RSS are stored in a fingerprint database. In the online stage, the user obtains the online RSS of the current position, the online RSS is matched with the offline RSS and select the best matched RPs as the estimated position. In WiFi fingerprint positioning experiments, some points with known positions are generally selected as the test points (TPs), the positioning accuracy of the system is evaluated according to the estimated and real positions of these TPs.

At present, the main research directions for fingerprint positioning are reducing the computational complexity and improving the positioning accuracy [4,5]. For the WiFi positioning, the positioning terminal is generally a battery-powered device with limited computing power [6]. For a large fingerprint database, searching all fingerprints needs a very large amount of computation, which seriously reduces the positioning efficiency. To reduce the computational complexity, the clustering-based fingerprinting methods are proposed; by the clustering, only the RPs of one cluster need be searched for each TP. The researchers have proposed many clustering methods, such as k-means [7], fuzzy C-means [8], mixture of Gaussian clustering [9], affinity propagation [10], and support vector machine clustering [11]. However, the clustering criteria used in the above RSS-based clustering methods are only the signal distance, which makes the clustering result unable to reflect the position distribution of RPs well and lead to the existence of many RPs with similar RSS, but distant positions, in a cluster. In [12], a coordinate-based clustering method is proposed to reduce the influence of AP deployment and improve the robustness of the system in dense cluttered indoor environments. In [13], a hybrid distance of signal distance and position distance is used as the clustering feature for clustering in the offline stage. For these methods, the clustering criterion of the offline stage is the position distance or the hybrid distance, while the cluster matching of the online stage is implemented by comparing the signal distance between the online RSS and each cluster center. Due to the inconsistency of the clustering criteria between the offline stage and online stage, the online cluster matching will become inaccurate. 

Fingerprint positioning methods can be divided into probabilistic methods and deterministic methods. Since the deterministic methods do not need to know the probability statistical model of the WiFi signal and are simpler to implement, they have been applied more widely. In the deterministic methods, the nearest neighbor-based methods are most frequently used; there are Nearest Neighbor (NN), K-Nearest Neighbor (KNN), and Weighted K-Nearest Neighbor (WKNN). Take the WKNN as an example: its working mechanism is to find K RPs which have the minimum signal distances from the online RSS, and weighted average the positions of the selected RPs according to their signal distances [14]. For the WKNN algorithm, the K-value and the distance measure are the two most important parameters, which have a significant impact on positioning accuracy. To select the optimal K-value, some researchers have presented the cluster-filtered WKNN methods, or adjust the K-value adaptively according to the changes of the indoor environment. In [15], the k-means algorithm groups nearest RPs according to their distance to the user and the closest group is used to calculate the position. In [16], the selected RPs are clustered based on their position distance, then the outlier RPs are filtered out and the subset with a larger number of RPs is reserved. An enhanced WKNN method is introduced in [17], and the number of nearest RPs is determined according to different location scenarios, such as corridors, halls, or rooms. A dynamic WKNN method is described in [18], which determines the optimal K-value based on the topological structure of the nearest RPs. For the distance measures in the WKNN methods, Euclidian distance [19,20] and Manhattan distance [21,22] are most widely employed to match the RSS fingerprints. To select the optimal distance measure, some researchers compare the positioning accuracy under different distance measures and improve the traditional Euclidian distance or Manhattan distance. In [21], the positioning performance with Euclidian distance and Manhattan distance are evaluated. In [23], to deal with the noise in the calculation of signal distance, different weights are assigned to the RSS fingerprint according to their importance. Ma [24] improves the Euclidean distance by introducing the standard deviation of RSS to smooth the signal fluctuation. Niu [22] proposes a weighted KNN method to assign different weights by defining the correlation coefficient between APs and achieve room-level localization accuracy. However, the nonlinear relationship between physical distance and differential RSS is not considered in the above methods. From the signal logarithmic attenuation model [25] we can find that the differential RSS should be a logarithm function of the physical distance [5]. Therefore, in the process of fingerprint matching and the weighted averaging of RP positions, using the traditional Euclidean distance or Manhattan distance will cause positioning errors. 

It is also pointed out that the fingerprint collection process is very time-consuming and laborious, which is a major disadvantage of the fingerprint method. Numerous researchers have aimed at the automatic construction of indoor fingerprints in a real scenario application, such as simultaneous localization and mapping (SLAM) [26,27], data interpolation [28,29], and crowdsourcing technology [30,31]. Nevertheless, the automatic construction of a fingerprint database may lead to the inaccuracy of fingerprint data, which is not the focus of this paper. To better demonstrate that our proposed method can achieve more appropriate fingerprint clustering and improve the positioning accuracy, our fingerprint collection work is still done manually.

To address the existing problems of WiFi fingerprint positioning, this paper proposes an improved WiFi positioning method based on fingerprint clustering and signal weighted Euclidean distance (SWED). The remainder of this paper is organized as follows: In Section 2, an overview of the proposed method is presented, and the proposed clustering and positioning algorithms are described in detail. In Section 3, the clustering and positioning experiments are performed, and the performance of the proposed method is evaluated. Finally, the conclusions are discussed in Section 4.

## 2. The Proposed WiFi Fingerprint Positioning Method

### 2.1. Overview of the Proposed WiFi Fingerprint Positioning Method

**Figure 1:**
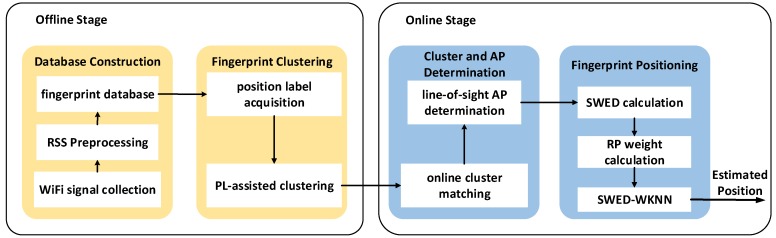
Overall structure of the proposed WiFi fingerprint positioning method.

The overall architecture of the proposed method is shown in Figure 1. In the offline stage, to reduce the influence of fading and shadowing on WiFi signal and make the RSS smoother, the original RSS observations are preprocessed. The traditional RSS-based clustering cannot well reflect the position distribution of RPs, to deal with this problem, we propose the position label-assisted (PL-assisted) clustering algorithm. We first implement the coordinate-based clustering using the k-means algorithm, and the clustering results are taken as the position labels of the RPs. Then, using the position labels as auxiliary information, all RPs are clustered by the Learning Vector Quantization (LVQ) algorithm. This process ensures that the clustering result is consistent with the position distribution of RPs and the position relationship between RPs. We theoretically analyze the distribution characteristic of WiFi signal and the positioning error of the traditional Euclidean distance-based WKNN (Euclidean-WKNN). To make the signal distance reflect the physical distance more accurately, we propose the SWED-based WKNN (SWED-WKNN) algorithm, which considers the nonlinear relationship between RSS and physical distance. Based on the overall size of each pair of RSS measurements, we assign weight to each differential RSS in the calculation of signal distance. In addition, to make our proposed SWED-WKNN algorithm more accurate and reasonable, we analyze the RSS distribution in actual environment. We define the line-of-sight AP (LOS-AP) based on the region of the cluster to which the TP belongs, and only the LOS-APs are used in the SWED calculation. The experiment results indicate that our proposed method can greatly improve the positioning accuracy. 

### 2.2. Received Signal Strength Preprocessing and Fingerprint Database

In an indoor environment, WiFi signals are significantly affected by fading and shadowing [32]. The fading is mainly caused by the multipath propagation of signals reflected in walls, rooms, and floors, which can cause strong fluctuations in RSS value. The shadowing such as the presence of a pedestrian between transmitter and receiver can considerably reduce the RSS value. The strong RSS would be mainly affected by fading, while the weak RSS may be affected by fading and shadowing or more factors [33]. Therefore, to reduce these influences, the RSS preprocessing method proposed in [33] is adopted in this paper. The method is to collect the original RSS observations within a certain period on a point, abandon the weakest RSS observations and take the average value of the strongest RSS observations as the RSS measurement. The preprocessed RSS is calculated by:

(1) $$RSS_{i}^{j}=\frac{1}{num.\;\max}\sum_{k\in num.\;\max}orig.\;RSS_{i,k}^{j}$$

where $RSS_{i}^{j}$ represents the preprocessed RSS measurement of the *j*th AP at the *i*th RP, and $orig.\;RSS_{i,k}^{j}$ represents the *k*th strongest original RSS observation. num. max is the number of the selected maximum RSS values and is set to 30 in this paper. In this way, the preprocessed RSS values are smoother, which is helpful for the following fingerprint clustering and positioning.

We divide the experimental area into grids and select the grid centers as the RPs. The format of the fingerprint database is shown as Table 1. The first column represents the ordinal number of the RPs, N and M are the number of the RPs and the APs, respectively. $x_{i}$ and $y_{i}$ are the coordinate of the *i*th RP, which is measured in the established two-dimensional coordinate system. As denoted in Equation (1), $RSS_{i}^{j}$ represents the preprocessed offline RSS measurement of the *j*th AP at the *i*th RP.

### 2.3. Position Label-Assisted Clustering Algorithm

The fingerprint clustering is to gather the RPs with high similarity and separate that with low similarity based on certain similarity criteria. For the existing fingerprint clustering algorithms, the fingerprint similarity criteria can be divided into position distance and signal distance, among which the Euclidean distance is widely used:

(2) $$d_{pos}\left(RP_{i},RP_{j}\right)=\sqrt{{\left(x_{i}-x_{j}\right)}^{2}+{\left(y_{i}-y_{j}\right)}^{2}}$$

(3) $$d_{sig}\left(RP_{i},RP_{j}\right)=\sqrt{\sum_{k=1}^{M}{\left(RSS_{i}^{k}-RSS_{j}^{k}\right)}^{2}}$$

where $d_{pos}\left(RP_{i},RP_{j}\right)$ and $d_{sig}\left(RP_{i},RP_{j}\right)$ represent the position and signal Euclidean distance between the *i*th RP and the *j*th RP, respectively. 

The issue of signal ambiguity and position ambiguity exists in the WiFi fingerprint positioning [34]. The signal ambiguity issue can be described as that two points are close to each other, while their signal distance is large. It is mainly caused by the fluctuation of RSS and can be reduced to some extent by averaging multi-epoch RSS signals [35]. However, the position ambiguity issue is more difficult to solve. It can be described as that two points are far from each other, while their signal distance is small. It may bring serious problem that a cluster may cover many RPs that are far apart but have small signal distance, and it will also cause inaccurate cluster matching in online stage. Therefore, to solve the position ambiguity issue, we consider both the position distribution and the signal distance of RPs, propose the PL-assisted clustering using LVQ algorithm. Different from the traditional clustering methods, LVQ assumes that the samples have labels and the labels can assist clustering [36]. 

To obtain the position labels, the coordinate-based clustering is first implemented based on the position distance between RPs, as indicated in Equation (2). Since the coordinate-based clustering is relatively simple, the most common k-means algorithm is adopted. Then we take the category that each RP belongs to in the coordinate-based clustering as its position label. The number of the coordinate-based clusters is predefined as P, that is, the value of the given position labels ranges from 1–P. The position labels are denoted by:

(4) $$L=\left\{L_{1},L_{2},\ldots ,L_{N}\right\}$$

where $L_{i}$ is the position label of the *i*-th RP and its value ranges from 1–P.

Our purpose is finding a set of the prototype vectors to characterize the clustering structure, each prototype vector defines a cluster. The number of the clusters is predefined as Q, which is also the number of the prototype vectors. In LVQ algorithm, the number of label types is not greater than the number of the clusters, that is, P≤Q. First, we need to select the Q offline RSS fingerprints as the Q initialized prototype vectors, so each prototype vector also has a label, which is the position label of the selected RP. To make the position labels of the prototype vectors traverse all the position labels, at least one RSS fingerprint is selected from each coordinate-based cluster as the initialized prototype vector. The prototype vectors and their position labels are denoted by:

(5) $$V=\left\{V_{1},V_{2},\ldots ,V_{Q}\right\}$$

(6) $$T=\left\{T_{1},T_{2},\ldots ,T_{Q}\right\}$$

where $V_{i}$ is the *i*th initialized prototype vector, $T_{i}$ is the position label of $V_{i}$ and its value ranges from 1–P. 

First, we randomly select a RP from the fingerprint database, calculate the signal Euclidean distances between the selected RP and the Q prototype vectors to find the prototype vector with the minimum distance. Then we determine whether the position label of the nearest prototype vector is the same as that of the selected RP. If the position labels are the same, the nearest prototype vector is considered to have the potential to be the cluster center of the selected RP, then update the prototype vector to make it closer to the selected RP; otherwise, the nearest prototype vector is considered to be potentially unsuitable as the cluster center of the selected RP, then update the prototype vector to make it farther away from the selected RP. 

For instance, for the position label $T_{j}$ of the nearest prototype vector $V_{j}$ the same as the position label $L_{i}$ of the selected $RSS_{i}$, this indicates that they both have similar RSS and belong to the same coordinate-based cluster. Therefore, $V_{j}$ is considered to have the potential to be the cluster center of $RSS_{i}$ and is updated to closer to $RSS_{i}$, denoted as:

(7) $$V_{j}^{\prime }=V_{j}+\alpha \left(RSS_{i}-V_{j}\right)$$

where α is the learning rate and ranges from 0 to 1, considering the convergence rate and the clustering effect of the algorithm, we set the learning rate to 0.1 empirically. The Euclidean distance between the updated prototype vector $V_{j}^{\prime }$ and $RSS_{i}$ is:

(8) $$\begin{array}{ll} d_{sig}\left(V_{j}^{\prime },RSS_{i}\right) & ={\left\|V_{j}+\alpha \left(RSS_{i}-V_{j}\right)-RSS_{i}\right\|}_{2}\\ & =\left(1-\alpha \right){\left\|V_{j}-RSS_{i}\right\|}_{2}\\ & =\left(1-\alpha \right)d_{sig}\left(V_{j},RSS_{i}\right) \end{array}$$

We can see that the updated prototype vector $V_{j}^{\prime }$ is closer to $RSS_{i}$. 

More importantly, for $T_{j}$ different from $L_{i}$, this indicates that although their signal distance is the smallest, their position distance may be large. Therefore, it is considered that $V_{j}$ may not be suitable as the cluster center of $RSS_{i}$ and it is updated to farther away from $RSS_{i}$, denoted as:

(9) $$V_{j}^{\prime }=V_{j}-\alpha \left(RSS_{i}-V_{j}\right)$$

(10) $$d_{sig}\left(V_{j}^{\prime },RSS_{i}\right)=\left(1+\alpha \right)d_{sig}\left(V_{j},RSS_{i}\right)$$

It should be noted that for given a large *P*-value, the types of position labels also increase. This will cause the labels of the prototype vectors to be scattered and increase the probability that the label of the prototype vectors is different from the label of the selected RP. Correspondingly, for those prototype vectors with minimum signal distance and acceptable position distance from the selected RP, they will be updated to distant the selected RP due to the difference of position labels. This will be adverse for the convergence and the clustering effect of the algorithm. Therefore, to obtain satisfactory clustering result in practical application, we need to evaluate the clustering under different *P*-values and select the optimal value.

After updating a prototype vector, we select the next RP and repeat the updating process, and the final prototype vectors can be obtained. Therefore, the RPs are classified into the cluster represented by the nearest prototype vector and all RPs are divided into the Q clusters. The stopping condition of the algorithm is to achieve the maximum number of iterations or the update range of the prototype vectors is very little. In the process of the online cluster matching, we calculate the signal Euclidean distances between the TP and all prototype vectors. Similar to the offline stage, the cluster represented by the nearest prototype vector is the cluster to which the TP belongs, denoted by:

(11) $$TP\in C_{i},\;if\;d_{sig}\left(TP,V_{i}\right)\leq d_{sig}\left(TP,V_{j}\right),\;j\in \left\{1,2,\ldots ,Q\right\}\;and\;j\neq i$$

Compared with the traditional RSS-based clustering, the PL-assisted clustering algorithm effectively utilizes the position distribution of RPs. In addition, compared with the traditional coordinate-based clustering and the hybrid distance-based clustering in [13], since the criteria of our offline clustering and online cluster matching are consistent, it reduces the misjudgment of online cluster matching. The pseudocode of Algorithm 1 is listed below. 


**Algorithm 1: The proposed PL-assisted clustering algorithm.**
**Input:** offline RSS fingerprints $\left\{RSS_{1},RSS_{2},\ldots ,RSS_{N}\right\}$;     position labels of offline RSS fingerprints $L=\left\{L_{1},L_{2},\ldots ,L_{N}\right\}$;     number of prototype vectors *Q*;     initialized prototype vector set $V=\left\{V_{1},V_{2},\ldots ,V_{Q}\right\}$;     position labels of prototype vectors $T=\left\{T_{1},T_{2},\ldots T_{Q}\right\}$;     learning rate α∈(0,1); 1: **repeat** 2:   randomly select an offline RSS fingerprint from the database; 3:   calculate the Euclidean distance between the selected $RSS_{i}$ and all prototype vectors: 4:   find the prototype vector $V_{j}$ closest to the selected $RSS_{i}$: 5:   **if**
$L_{i}=T_{j}$ 6:    $V_{j}^{\prime }=V_{j}+\alpha \left(RSS_{i}-V_{j}\right)$; 7:   **else** 8:    $V_{j}^{\prime }=V_{j}-\alpha \left(RSS_{i}-V_{j}\right)$; 9:   **end** 10:    the prototype vector $V_{j}$ is updated as $V_{j}^{\prime }$; 11:    return to line 2; 12: **until** achieve the maximum number of iterations or the update range of the prototype vectors is very little; **Output:** the final prototype vector $\left\{V_{1},V_{2},\ldots ,V_{Q}\right\}$;

### 2.4. Signal Weighted Euclidean Distance-Based Weighted K-Nearest Neighbor Algorithm

Before describing our proposed positioning method, we first theoretically analyze the distribution characteristic of WiFi signals and the positioning error of the traditional Euclidean-WKNN algorithm. Many WiFi signal attenuation models are summarized in previous work [37,38], such as log-distance, multi-slope, COST231, and International Telecommunication Union (ITU) models, which have been widely used to construct and update fingerprint database automatically. For convenience, the log-distance model is adopted for the theoretical analysis in this paper. As indicated in [25], the classical WiFi signal attenuation model is expressed by:

(12) $$P_{L}\left(d_{i}\right)=P_{L}\left(d_{0}\right)-10\eta {log}_{10}\left(\frac{d_{i}}{d_{0}}\right)+\chi_{\sigma }$$

where $P_{L}\left(d_{i}\right)$ represents the RSS at the point which has a distance $d_{i}$ to the AP. $d_{0}$ is the reference distance and usually set to 1 m. η is the path loss exponent and $\chi_{\sigma }$ is a Gaussian random variable with standard deviation σ. Therefore, the physical distance $d_{i}$ and the differential physical distance Δd can be easily calculated by:

(13) $$d_{i}=d_{0}10^{\left(\frac{P_{L}\left(d_{0}\right)-P_{L}\left(d_{i}\right)}{10\eta }\right)}$$

(14) $$\Delta d=d_{i}-d_{j}=d_{0}10^{\left(\frac{P_{L}\left(d_{0}\right)-P_{L}\left(d_{i}\right)}{10\eta }\right)}-d_{0}10^{\left(\frac{P_{L}\left(d_{0}\right)-P_{L}\left(d_{j}\right)}{10\eta }\right)}$$

Based on the simulation data obtained by Equations (13) and (14), we analyze the relationship between RSS and physical distance. According to the results reported in [39], the path loss exponent η is set to 2.76, the reference distance $d_{0}$ is 1 m and $P_{L}\left(d_{0}\right)$ is −31.7 dBm. As shown in Table 2, for the same differential RSS value (ΔRSS is equal to 1 dBm), the differential physical distances (Δd) under different RSS values are different. A pair of small RSS values is accompanied by a large differential physical distance, the relationship between the differential RSS and differential distance is nonlinear. 

We continue to analyze the positioning error of the traditional Euclidean-WKNN based on simulation data. As shown in Table 3, to understand it intuitively and simply, we only consider two APs, two RPs and five TPs in the one-dimensional coordinate system, and they are all on the same floor and in a straight line. 

For a pair of RP and TP, the differential RSS values from two APs are different. Additionally, the ratio of the signal Euclidean distances between the TP and different RPs is not consistent with that of the physical distances. For instance, TP3 has the same physical distance (6 m) from RP1 and RP2, but its signal Euclidean distances from the two RPs are different (11 dBm for RP1 and 6.47 dBm for RP2). This is because, for a pair of RP and TP, the traditional Euclidean-WKNN only considers the size of the differential RSS but not the overall RSS size of a pair of RP and TP, which leads to errors in measuring physical distance. Therefore, although the simulation data can be considered as the positioning in an ideal environment without interference, multipath and other factors, there are still positioning errors for the traditional Euclidean-WKNN. 

The above theoretical analysis shows that the nonlinear relationship between the differential RSS and physical distance should be considered in WKNN algorithm. Therefore, we propose the signal weighted Euclidean distance based WKNN (SWED-based WKNN) algorithm to improve the positioning accuracy. As mentioned before, in the initial stage of positioning, we calculate the signal Euclidean distances between each TP and all prototype vectors using Equation (11), and the cluster represented by the nearest prototype vector is the cluster to which the TP belongs. Therefore, only the RPs within that cluster are searched for each TP.

We first use the average RSS value to measure the overall size of each pair of RSS for a RP and a TP, then we calculate the difference between the average RSS value and $P_{L}\left(d_{0}\right)$, denoted by:

(15) $$DAR_{i}^{j}=abs\left(P_{L}^{j}\left(d_{0}\right)-avg\left(RSS_{i}^{j},RSS^{j}\right)\right)$$

where $RSS_{i}^{j}$ is the offline RSS measurement of the *j*th AP at the *i*th RP, $RSS^{j}$ is the online RSS measurement of the *j*th AP at the TP, $P_{L}^{j}\left(d_{0}\right)$ is the RSS value of the *j*th AP at reference distance. avg() and abs() are the average value function and the absolute value function, respectively. 

As the analysis in Table 2, given the same differential RSS value, a pair of small RSS values is accompanied by a large differential physical distance. A pair of small RSS values means a small $avg\left(RSS_{i}^{j},RSS^{j}\right)$ and a large $DAR_{i}^{j}$. Therefore, to balance the differential RSS and the physical distance, we assign a large weight to a large $DAR_{i}^{j}$:

(16) $$\omega_{i}^{j}=\frac{DAR_{i}^{j}}{\sum_{j=1}^{m}DAR_{i}^{j}}=\frac{abs\left(P_{L}^{j}\left(d_{0}\right)-avg\left(RSS_{i}^{j},RSS^{j}\right)\right)}{\sum_{j=1}^{m}abs\left(P_{L}^{j}\left(d_{0}\right)-avg\left(RSS_{i}^{j},RSS^{j}\right)\right)}$$

Then, we assume that the path loss exponents of all APs are the same and assign weights to the differential RSS of different APs. Thus, the SWED is calculated by:

(17) $$SWED\left(RP_{i},TP\right)=\sqrt{\frac{1}{m}\sum_{j=1}^{m}\omega_{i}^{j}{\left(RSS_{i}^{j}-RSS^{j}\right)}^{2}}$$

where $SWED\left(RP_{i},TP\right)$ is the SWED between the i-th RP and the TP, $\omega_{i}^{j}$ represents the weight of the differential RSS of the j-th AP. *m* is the number of detected same APs, since the number of same APs at different RPs is varying, the signal distance is averaged by *m* to ensure the fairness of distance comparison. Finally, K nearest RPs with the minimum SWEDs are selected, the weights of the nearest RPs’ coordinates are calculated by:

(18) $$\lambda_{i}=\frac{\frac{1}{SWED\left(RP_{i},TP\right)}}{\sum_{i=1}^{K}\frac{1}{SWED\left(RP_{i},TP\right)}}$$

The position is estimated by:

(19) $$\left(x,y\right)=\sum_{i=1}^{K}\lambda_{i}\left(x_{i},y_{i}\right)$$

where $\lambda_{i}$ is the weight of the *i*th RP, (x,y) and $\left(x_{i},y_{i}\right)$ are the estimated position and the position of the *i*th RP, respectively. Obviously, the RP with a large SWED is assigned a small weight, which can reduce the contribution of the RPs away from the TP. 

**Figure 2:**
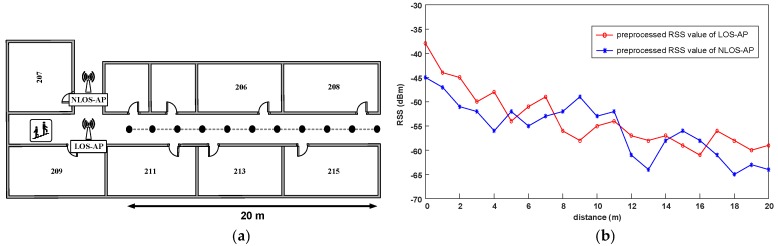
Analysis of the RSS distribution in actual experimental site. (**a**) The schematic diagram of the experimental filed; and (**b**) the RSS distribution of the LOS-AP and the NLOS-AP over distance.

It should be noted that for the practical positioning, fading, shadowing, and dynamic environment significantly make the RSS fluctuation [24]. Although we have preprocessed the original RSS observations, these effects cannot be eliminated completely. Therefore, we analyze the RSS distribution in actual experimental environment. According to whether there is a wall or other obstacles between the AP and the receiver, we divide the APs into the line-of-sight APs (LOS-APs) and the non-line-of-sight APs (NLOS-APs). For convenience, for a position point, we consider the APs in the same corridor as the LOS-APs of this point, and the others as the NLOS-APs. As shown in Figure 2a, we employ a LOS-AP and a NLOS-AP, select 21 points with different distance from the APs. The total span of the points is 20 m, the interval between adjacent points is 2 m and the signal collection time is three minutes at each point. Using the RSS preprocessing method in Section 2.2, we obtain the processed RSS at different distances. 

From Figure 2b we can see that, because of the reflection and refraction of the signals, the WiFi signals show strong fluctuation. Especially the RSS distribution of the LOS-AP is more complex and unpredictable, and the signal curve of the LOS-AP is also not smooth and has many mutations. However generally speaking, compared with the NLOS-AP, the RSS distribution of the LOS-AP is basically consistent with the change of distance, that is, the signal intensity becomes weaker with the increase of distance and the signal attenuation rate is relatively faster in the distance closer to the AP. Therefore, it can be predicted that using different APs may have a significant impact on the performance of our proposed positioning method.

The experimental site will be introduced in the next section of the experimental setup.

**Figure 3:**
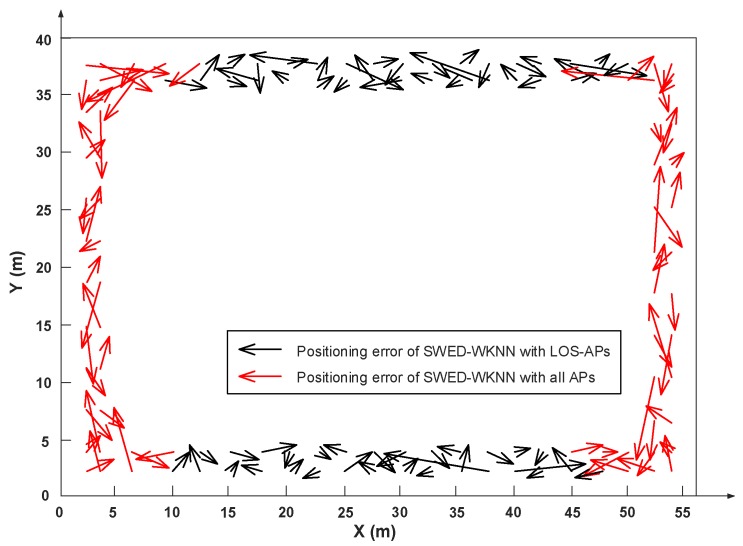
Comparison of the positioning error of the SWED-WKNN method with different APs.

To confirm our speculation, we compare the positioning accuracy of our proposed algorithm with the LOS-APs and the NLOS-APs in the experimental field 1, which is introduced in the next section of the experimental setup and 120 TPs are selected. We use the error vectors to show the positioning performance more intuitively. As shown in Figure 3, the arrows point from the actual positions to the corresponding estimated positions, black arrows indicate the positioning errors of the SWED-WKNN with only LOS-APs, and red arrows indicate the positioning errors of the SWED-WKNN with all detected APs. We can see that the proposed SWED-WKNN method has larger positioning errors when all the APs are used. This is because the signal propagation path from the NLOS-APs is more complex, its RSS distribution may not be consistent with the signal attenuation model. Additionally, the values of the RSS from the LOS-APs and the NLOS-APs are confused. In the SWED calculation for a pair of RP and TP, the weights calculated by the RSS average values from different APs will become inaccurate, resulting in errors in the obtained SWED. To make our proposed SWED-WKNN method effective, in this paper, the positions of all APs are known and only the LOS-APs are used. Our solutions are as follows:

1. In the initial stage of positioning, we determine which cluster the TP belongs to using Equation (11). If the cluster is located at the corner of the experimental field, the traditional Euclidean-WKNN algorithm is used for positioning. This is because, for the RPs and TPs in the corner-clusters, their LOS-APs may be in either of the two corridors and are difficult to determine.
2. If the cluster the TP belongs to is not located at the corner of the experimental field, we consider the APs in the same corridor where this cluster is located as the LOS-APs. Then, we only use the RSS from these LOS-APs in our proposed SWED-WKNN algorithm.

Therefore, it should be pointed out that our proposed SWED-WKNN algorithm is useful for the fingerprint positioning with LOS-APs or when the RSS distribution is basically consistent with the WiFi signal attenuation model, and it is not suitable for positioning with the NLOS-APs or the multi-floor APs.

## 3. Experiments and Results

### 3.1. Experimental Setup

**Figure 4:**
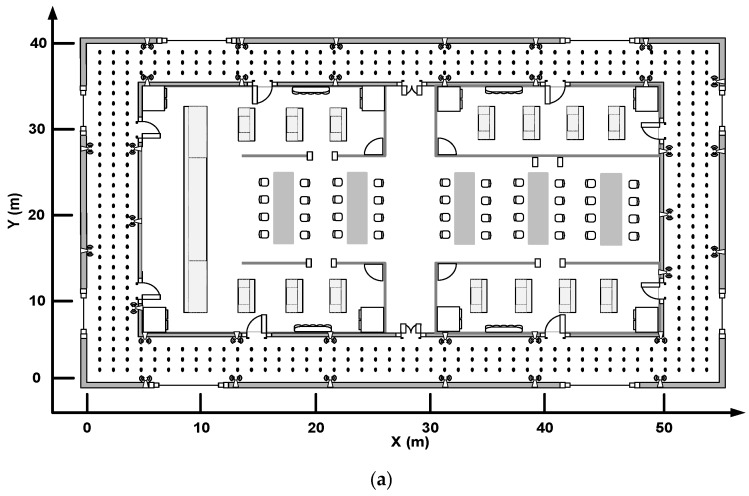

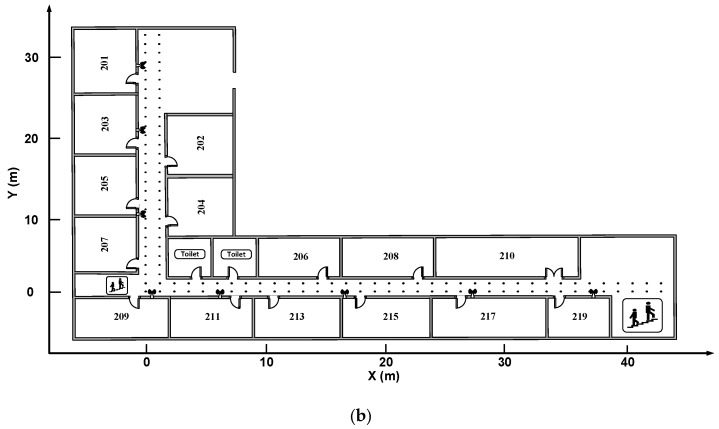
Indoor fingerprint positioning experiment environment. (**a**) Experimental field 1 on the fifth floor of the library building; and (**b**) experimental field 2 on the second floor of the academic building.

To demonstrate the applicability of our proposed method in different indoor environments, the proposed method is tested by experiments conducted in two experimental fields. As shown in Figure 4, the black points represent the RPs and the distance between adjacent RPs is 1.2 m. Experimental field 1 is situated on the fifth floor of a library building, where 426 RPs and 35 APs are deployed. Experimental field 2 is situated on the second floor of an academic building, where 128 RPs and eight APs are deployed. The WiFi RSS are collected by Xiaomi MIX2 smartphone and the signal sampling frequency is 1 Hz. For each RP and TP, the RSS collection time is two minutes and 30 maximum original RSS observations are averaged as the processed RSS measurements in the offline and online stages. 

### 3.2. Result of Clustering Experiment

In this section, to evaluate the performance of different clustering methods, the Davies–-Bouldin Index (DBI) [40] is introduced. DBI is a clustering validity index which can help us quantify the clustering effect based on the clustering data, and we can make a judgment according to the actual meaning of the data. To obtain the DBI value, the intra-cluster distance and inter-cluster distance need to be calculated first, which represent the dispersion degree of the RPs, calculated by:

(20) $$intra.\;d_{sig}\left(C_{i}\right)=\frac{2}{\left|C_{i}\right|\left(\left|C_{i}\right|-1\right)}\sum_{1\leq i<j\leq \left|C_{i}\right|}d_{sig}\left(RP_{i},RP_{j}\right)$$

(21) $$intra.\;d_{pos}\left(C_{i}\right)=\frac{2}{\left|C_{i}\right|\left(\left|C_{i}\right|-1\right)}\sum_{1\leq i<j\leq \left|C_{i}\right|}d_{pos}\left(RP_{i},RP_{j}\right)$$

(22) $$inter.\;d_{sig}\left(C_{i},C_{j}\right)=d_{sig}\left(\mu_{sig,i},\mu_{sig,j}\right)$$

(23) $$inter.\;d_{pos}\left(C_{i},C_{j}\right)=d_{pos}\left(\mu_{pos,i},\mu_{pos,j}\right)$$

where $intra.\;d_{sig}\left(C_{i}\right)$ and $intra.\;d_{pos}\left(C_{i}\right)$ represent the intra-cluster distances of the *i*th cluster in the signal domain and the position domain, respectively. $inter.\;d_{sig}\left(C_{i},C_{j}\right)$ and $inter.\;d_{pos}\left(C_{i},C_{j}\right)$ represent the inter-cluster distances between the *i*th cluster and the *j*th cluster in the signal domain and the position domain, respectively. $\left|C_{i}\right|$ is the number of the RPs in the *i*th cluster, $\mu_{sig,i}$ and $\mu_{pos,i}$ are the signal center and position center of the *i*th cluster, respectively. 

Based on the intra-cluster and inter-cluster distances of the clusters, the signal-domain DBI, the position-domain DBI and the hybrid DBI are calculated by:

(24) $$DBI_{sig}=\frac{1}{Q}\sum_{i=1}^{Q}\underset{j\neq i}{max}\left(\frac{intra.\;d_{sig}\left(C_{i}\right)+intra.\;d_{sig}\left(C_{j}\right)}{inter.\;d_{sig}\left(C_{i},C_{j}\right)}\right)$$

(25) $$DBI_{pos}=\frac{1}{Q}\sum_{i=1}^{Q}\underset{j\neq i}{max}\left(\frac{intra.\;d_{pos}\left(C_{i}\right)+intra.\;d_{pos}\left(C_{j}\right)}{inter.\;d_{pos}\left(C_{i},C_{j}\right)}\right)$$

(26) $$DBI_{hyb}=\sqrt{DBI_{sig}\cdot DBI_{pos}}$$

where Q is the number of the clusters, $DBI_{sig}$, $DBI_{pos}$ and $DBI_{hyb}$ are the signal-domain DBI, the position-domain DBI and the hybrid DBI, respectively. As indicated in Equations (24) and (25), for each cluster, the maximum ratio value of the intra-cluster distance to the inter-cluster distance is selected, and the DBI is obtained by averaging these maximum ratio values. Generally, the maximum ratio value comes from the adjacent clusters, which is most important for evaluating clustering performance. As indicated in Equations (26), since the hybrid DBI is the square of product of the signal-domain DBI and the position-domain DBI, it can reflect both the signal relationship and position relationship of the RPs after the clustering. Clearly, a small DBI value means a good clustering performance, that is, the intra-cluster distances are small and the inter-cluster distances are large. 

Table 4 lists three DBI values of the proposed PL-assisted clustering algorithm under different P-values for a given fixed Q-value. The learning rate is set to 0.1 and the maximum number of iterations is set to 5000. As mentioned in the Section 2.3, the P-value is not greater than the Q-value. For the same Q-value we can see that, as the P-value is close to Q-value, some position-domain DBI values decrease and some signal-domain DBI values increase, while some other position-domain DBI (signal-domain DBI) values may decrease (increase) first and then increase (decrease). Similarly, the variation of hybrid DBI values is also not completely consistent with the variation of P-value. As explained in Section 2.3, for given a large number of types of position labels, the position distribution of RPs is clearer. However, this also increase the probability that the label of the prototype vectors is different from the label of the selected RP. Correspondingly, for those prototype vectors with minimum signal distance and acceptable position distance from the selected RP, they will be updated to distant the selected RP due to the difference of position labels. Therefore, we select the P-value with the minimum hybrid DBI value for the comparison with other clustering method.

Similar to the proposed algorithm, k-means is also a prototype-based clustering algorithm, which is widely used in WiFi fingerprint clustering. Therefore, we choose k-means algorithm for comparison and the clustering criterion of k-means is also the signal Euclidean distance. 

**Figure 5:**
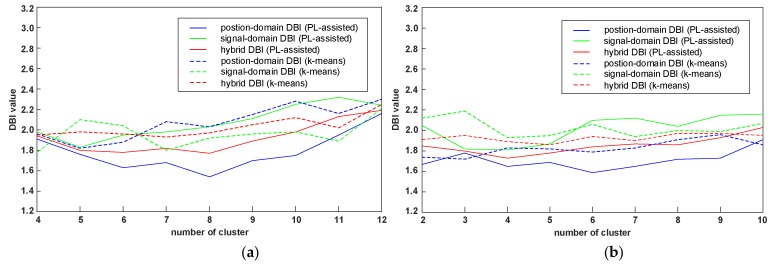
The DBI value comparison between the proposed PL-assisted clustering algorithm and the k-means algorithm under different number of the clusters. (**a**) The DBI value comparison in experimental field 1; and (**b**) the DBI value comparison in experimental field 2.

According to the number of RPs and the experimental area, the Q-values from 4–12 are considered in experimental field 1 and from 2–10 in experimental field 2. Figure 5 shows the position-domain DBI, the signal-domain DBI, and the hybrid DBI value comparison between the proposed clustering algorithm and k-means algorithm under different Q-values. As shown in Figure 5a, for the proposed algorithm in the experimental field 1, the minimum values of the position-domain DBI, the signal-domain DBI, and the hybrid DBI are 1.54, 1.83, and 1.77 when the Q-values equal 8, 5, and 8, respectively. Accordingly, that of the k-means are 1.82, 1.78, and 1.93 when the Q-values equal 5, 4, and 7, respectively. As shown in Figure 5b, for the proposed algorithm in the experimental field 2, the minimum values of the position-domain DBI, the signal-domain DBI and the hybrid DBI are 1.59, 1.81 and 1.73 when the Q-values equal 6, 4 and 4, respectively. Accordingly, that of the k-means are 1.72, 1.93 and 1.87 when the Q-values equal 3, 4, and 5, respectively. It can be concluded that the position-domain DBI values of the proposed algorithm are smaller than that of the k-means, while the signal-domain DBI values are larger than the k-means for some Q-values. Compared with the signal-domain DBI, we are more concerned about the position-domain DBI, because for the position estimation, our ultimate purpose is to find the RPs closest to the position of the TP. The position labels are used as the supervised information to assist clustering, and they can reflect the position distribution of the RPs. Thus, some prototype vectors with the nearest signal distance, but inconsistent labels, do not serve as the cluster center, which, to some extent, leads to the increase of the signal-domain DBI value. Nevertheless, the hybrid DBI values of the proposed algorithm are still smaller than that of the k-means. Considering both the position-domain and the signal-domain, it means that the proposed clustering algorithm has a smaller ratio value of the intra-cluster distance to the inter-cluster distance, and the clustering performance outperforms the k-means algorithm.

**Figure 6:**
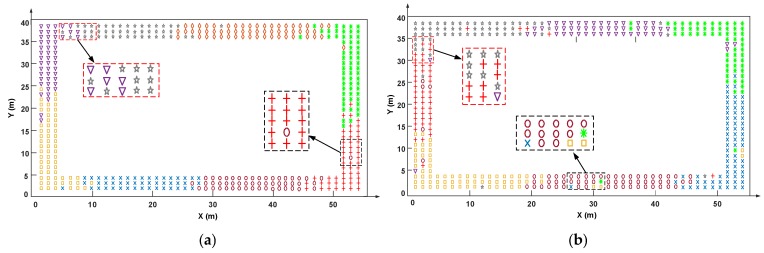

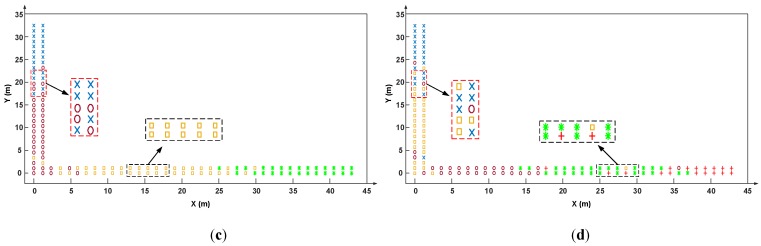
The clustering result comparison between the proposed PL-assisted clustering algorithm and the k-means algorithm. (**a**) The clustering result of the proposed algorithm in experimental field 1, the number of clusters is 8; (**b**) the clustering result of the k-means algorithm in experimental field 1, the number of clusters is 7; (**c**) the clustering result of the proposed algorithm in experimental field 2, the number of clusters is 4; and (**d**) the clustering result of the k-means algorithm in experimental field 2, the number of clusters is 5.

Figure 6 intuitively shows the clustering result comparison between the proposed algorithm and the k-means algorithm under the number of the clusters with the minimum hybrid DBI values in experimental fields 1 and 2; different colors and shapes denote different clusters. The red box denotes the RPs in the boundary region of adjacent clusters, and the black box denotes the RPs in the middle region of the cluster. We can see, for the two algorithms, there are both some RPs confused in the boundary region of adjacent clusters. This is because WiFi signals are influenced by a complex channel environment, which makes the RSS distribution in the boundary region not clear enough, resulting in the confusion of the RPs’ distribution. However, as shown in the black boxes, compared with the k-means, the RPs clustered by the proposed algorithm can be divided more clearly, and there are fewer outliers in the middle region of the cluster, these outliers are the RPs with similar RSS but distant positions. 

**Figure 7:**
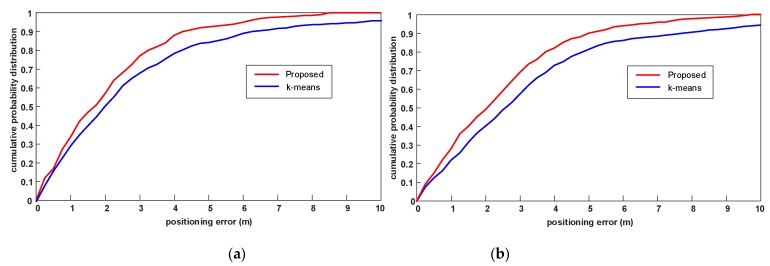
The effect of the PL-assisted clustering algorithm and k-means algorithm on positioning accuracy. (**a**) The positioning accuracy comparison in experimental field 1; and (**b**) the positioning accuracy comparison in experimental field 2.

Now we evaluate the effect of the different clustering algorithms on positioning accuracy. We select 120 points and 100 points as the TPs in experimental field 1 and experimental field 2, respectively. For convenience, we use the Euclidean-WKNN algorithm for the position estimation in this experiment and compare the positioning accuracy in terms of cumulative probability distribution. As shown in Figure 7, because of the reduction of those RPs in the cluster whose positions are far apart, the positioning error with the proposed PL-assisted clustering algorithm is smaller. It can also be concluded that better clustering results can produce better positioning performance. 

### 3.3. Result of Positioning Experiment

To evaluate the performance of our proposed positioning algorithm, we compare the positioning accuracy of three positioning algorithms under different distance measures (the traditional Euclidean-WKNN and Manhattan-WKNN, and the proposed SWED-WKNN). From the clusters that are not located the corners of the two experimental fields, we select 60 TPs in experimental field 1 and 50 TPs in experimental field 2. For different algorithms, the APs used for positioning are all the LOS-APs and the values of K are all set to 4. 

**Figure 8:**
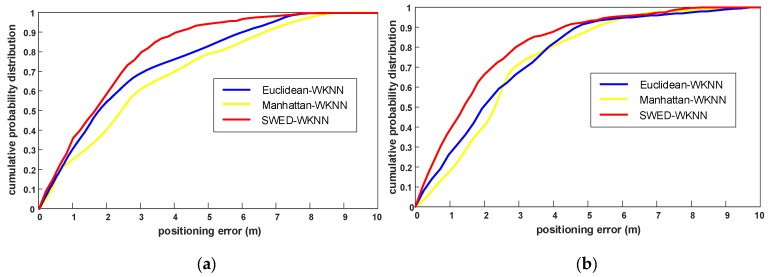
The comparison of cumulative probability distributions of the positioning errors of the traditional Euclidean-WKNN and Manhattan-WKNN, and the proposed SWED-WKNN. (**a**) The cumulative probability distributions of the positioning errors in experimental field 1; and (**b**) the cumulative probability distributions of the positioning errors in experimental field 2.

As shown in Figure 8, we can see that using the LOS-APs, the positioning accuracy of the proposed SWED-WKNN algorithms outperforms the other two algorithms. Table 5 lists the positioning error statistics of three algorithms. Compared with the Euclidean-WKNN and Manhattan-WKNN, for the positioning in experimental field 1, the mean error improvement of SWED-WKNN is 9.6% and 25.6%, and the RMSE improvement is 12.9% and 32.3%. For the positioning in experimental field 2, the mean error improvement of SWED-WKNN is 19.1% and 24.7%, and the RMSE improvement is 22.4% and 28.3%. The positioning accuracy method can satisfy the requirements of indoor positioning. 

To show the advantages of the proposed SWED-WKNN algorithm more intuitively, Table 6 lists the comparison between the SWED-WKNN and the traditional Euclidean-WKNN on the selected nearest RPs, the physical distances and the positioning errors. The nearest RPs selected by SWED and Euclidean distance are arranged according to the size of their signal distances, respectively. We can see that compared with the traditional Euclidean distance, the physical distances between the TP and the nearest RPs are more consistent with the SWED. In other words, the nearest RPs are basically sorted in the same order as the SWED. This should be attributed to the introduction of weights in calculating signal distances and the proposed SWED-WKNN can reflect the physical distance between the TP and the RPs more accurately, so that the positioning error can be reduced.

## 4. Conclusion and Future Work

This paper presents an improved WiFi positioning method based on fingerprint clustering and signal weighted Euclidean distance. The method is intended to cope with the issue of the nonlinear relationship between the RSS and the physical distance. The position distribution information of RPs is exploited to improve the clustering performance. Meanwhile, to make the signal distance reflect the physical distance better, we assign weights to different differential RSS in the calculation of the signal distance. The performance of this method is evaluated by the experiments in two typical office buildings. The experimental results demonstrate that the proposed PL-assisted clustering algorithm outperforms the traditional k-means algorithm, and the proposed SWED-WKNN algorithm outperforms the traditional Euclidian-WKNN and Manhattan-WKNN algorithms for the fingerprint positioning with the line-of-sight APs. For future work, to enhance the environmental adaptability of fingerprint positioning and solve the problem of fingerprint collection, we will research the automatic construction and update of a fingerprint database. In addition, we will further solve the problems in clustering, such as eliminating the outliers of the cluster and integrating the confusing RPs in regions of two adjacent clusters.

## Figures and Tables

**Table 1:** The format of the fingerprint database.

RPs	X	Y	$AP_{1}$	$AP_{2}$	$AP_{3}$	…	$AP_{M}$
1	$x_{1}$	$y_{1}$	$RSS_{1}^{1}$	$RSS_{1}^{2}$	$RSS_{1}^{3}$	…	$RSS_{1}^{M}$
2	$x_{2}$	$y_{2}$	$RSS_{2}^{1}$	$RSS_{2}^{2}$	$RSS_{2}^{3}$	…	$RSS_{2}^{M}$
...	...	...	...	...	...	...	...
*N*	$x_{N}$	$y_{N}$	$RSS_{N}^{1}$	$RSS_{N}^{2}$	$RSS_{N}^{3}$	…	$RSS_{N}^{M}$

**Table 2:** Analysis of the relationship between differential RSS value and differential physical distance based on simulation data.

RSS (dBm)	d (m)	ΔRSS (dBm)	Δd (m)
−30	0.8678		
−31	0.9433	1	0.0755
−32	1.0253	1	0.0821
−33	1.1146	1	0.0892
…	…		…
−51	5.0035	1	0.4005
−52	5.4389	1	0.4353
−53	5.9121	1	0.4732
…	…		…
−71	26.5407	1	2.1244
−72	28.8499	1	2.3092
−73	31.3601	1	2.5101

**Table 3:** Analysis of the positioning error of Euclidean-WKNN based on simulation data.

	Actual Position	RSS of AP1 and AP2	Differential RSS from RP1	Differential RSS from RP2	SED from RP1 and RP2	Estimated Position	Position Error
AP1	0						
AP2	5						
RP1	10	(−59.30, −50.99)					
RP2	22	(−68.75, −65.66)					
TP1	12	(−61.49, −55.02)	(2.19, 4.03)	(7.26, 10.64)	(4.59, 12.88)	13.15	1.15
TP2	14	(−63.33, −58.04)	(4.03, 7.05)	(5.42, 7.62)	(8.12, 9.35)	15.58	1.58
TP3	16	(−64.93, −60.44)	(5.63, 9.45)	(3.82, 5.22)	(11.00, 6.47)	17.56	1.56
TP4	18	(−66.35, −62.44)	(7.05, 11.45)	(2.40, 3.22)	(13.45, 4.02)	19.24	1.24
TP5	20	(−67.61, −64.16)	(8.31, 13.17)	(1.14, 1.50)	(15.57, 1.88)	20.70	0.70

**Table 4:** The DBI values of the proposed PL-assisted clustering algorithm under different P-values for a given Q-value.

Q-Value	Q = 8 (Experimental Field 1)							Q = 6 (Experimental Field 2)				
P-Value	P = 2	P = 3	P = 4	P = 5	P = 6	P = 7	P = 8	P = 2	P = 3	P = 4	P = 5	P = 6
Position DBI	2.5531	2.2641	2.0396	1.8966	1.5406	1.6375	1.4746	2.5967	2.1208	1.7381	1.5892	1.7546
Signal DBI	1.8964	1.9932	1.9776	2.1580	2.0334	2.0592	2.4201	1.7643	1.8364	2.0163	2.1341	2.2069
Hybrid DBI	2.2004	2.1244	2.0084	2.0231	1.7715	1.8362	1.8891	2.1404	1.9735	1.8720	1.8416	1.9678

**Table 5:** The positioning error statistics of the traditional Euclidean-WKNN and Manhattan-WKNN, and the proposed SWED-WKNN.

Method	Positioning Error in Experimetal Field 1 (m)				Positioning Error in Experimetal Field 2 (m)			
	50% Error	75% Error	Mean Error	RMSE	50% Error	75% Error	Mean Error	RMSE
Euclidean-WKNN	1.80	3.77	2.61	3.25	1.95	3.51	2.83	3.53
Manhattan-WKNN	2.51	4.59	3.17	4.18	2.37	3.26	3.04	3.82
SWED-WKNN	1.67	2.86	2.36	2.83	1.43	2.54	2.29	2.74

**Table 6:** Nearest RPs selection, physical distances between nearest RPs and TP, and positioning error using the proposed SWED-WKNN and the traditional Euclidean-WKNN.

Method		Position of TPs	Position of Selected Nearest RPs				Physical Distance Between TP and Nearest RPs				Estimated Position	Error
			1st	2nd	3rd	4th	1st	2nd	3rd	4th		
Experimental field 1	SWED-WKNN	(18.6, 3.0)	(19.2, 2.4)	(18.0, 1.2)	(16.8, 1.2)	(21.6, 1.2)	0.85	1.90	2.55	3.50	(18.91, 2.15)	1.29
		(29.4, 1.8)	(28.8, 3.6)	(32.4, 1.2)	(24.0, 3.6)	(37.2, 2.4)	1.90	3.50	5.70	7.82	(29.72, 3.34)	1.53
		(19.8, 39.0)	(19.2, 37.2)	(16.8, 39.6)	(15.6, 38.4)	(16.8, 37.2)	1.90	3.06	4.24	3.50	(18.03, 38.48)	1.89
		(42.6, 37.8)	(46.8, 38.4)	(48.0, 37.2)	(40.8, 37.2)	(50.8, 39.6)	4.24	5.43	1.90	8.40	(40.26, 38.76)	2.56
		(31.8, 37.8)	(28.8, 39.6)	(36.0, 39.6)	(36.0, 39.6)	(25.2, 37.2)	3.06	4.24	4.24	6.63	(31.20, 38.89)	1.30
	Euclidean-WKNN	(18.6, 3.0)	(24.0, 1.2)	(18.0, 1.2)	(12.6, 1.2)	(21.6, 2.4)	5.70	1.90	6.26	3.06	(19.05, 1.50)	1.57
		(29.4, 1.8)	(26.4, 3.6)	(20.4, 3.6)	(36.0, 3.6)	(36.0, 2.4)	3.50	9.18	6.84	6.63	(30.63, 3.37)	1.92
		(19.8, 39.0)	(20.4, 37.2)	(18.0, 37.2)	(15.6, 39.6)	(18.0, 39.6)	1.90	2.55	4.24	1.90	(17.17, 38.12)	2.85
		(42.6, 37.8)	(42.0, 37.2)	(39.6, 38.4)	(40.8, 38.4)	(38.4, 39.6)	0.85	3.06	1.90	4.57	(46.65, 38.09)	4.01
		(31.8, 37.8)	(31.2, 39.6)	(33.6, 39.6)	(28.8, 36.0)	(25.2, 37.2)	1.90	2.55	3.50	6.63	(29.72, 38.15)	2.12
Experimental field 2	SWED-WKNN	(7.8, 0.6)	(7.2, 2.4)	(6.0, 2.4)	(7.2, 0.0)	(4.8, 1.2)	1.90	2.55	0.85	3.06	(6.38, 1.45)	1.74
		(9.6, 1.2)	(9.0, 1.2)	(9.0, 0.0)	(13.8, 1.2)	(3.6, 1.2)	0.60	1.34	4.20	6.00	(8.85, 0.92)	0.81
		(11.4, 1.2)	(14.4, 1.2)	(10.8, 1.2)	(10.8, 0.0)	(13.2, 0.0)	3.00	0.60	1.34	2.16	(12.36, 0.63)	1.08
		(28.2, 0.6)	(26.4, 0.0)	(31.2, 1.2)	(25.2, 1.2)	(22.8, 1.2)	1.90	3.06	3.06	5.43	(26.45, 0.61)	1.76
		(39.0, 1.2)	(40.8, 1.2)	(36.0, 0.0)	(42.0, 0.0)	(31.2, 1.2)	1.80	3.23	3.23	7.80	(37.53, 0.66)	1.63
	Euclidean-WKNN	(7.8, 0.6)	(7.2, 1.2)	(4.8, 1.2)	(10.2, 1.2)	(12.6, 0.0)	0.85	3.06	2.47	4.84	(8.71, 0.90)	0.95
		(9.6, 1.2)	(12.6, 2.4)	(15.0, 0.0)	(7.8, 1.2)	(6.6, 2.4)	3.23	5.53	1.80	3.23	(11.52, 1.55)	1.54
		(11.4, 1.2)	(10.8, 1.2)	(10.8, 0.0)	(16.8, 0.0)	(14.4, 1.2)	0.60	1.34	5.53	3.00	(13.29, 0.60)	1.89
		(28.2, 0.6)	(26.4, 1.2)	(24.0, 0.0)	(31.2, 1.2)	(20.4, 1.2)	1.90	4.24	3.06	7.82	(25.58, 0.94)	2.72
		(39.0, 1.2)	(40.8, 1.2)	(38.4, 0.0)	(32.4, 0.0)	(42.0, 0.0)	1.80	1.34	6.71	3.23	(36.47, 0.323)	2.81

## References

[1] Melania S., Valérie R., Gérard L. (2013). Motion mode recognition and step detection algorithms for mobile phone users. Sensors.

[2] Weiser M. (1999). The computer for the 21st Century. ACM SIGMOBILE Mobile Computing and Communications Review.

[3] Shin B.J., Lee K.W., Choi S., Kim J., Lee W.J., Kim H.S. Indoor WiFi positioning system for Android-based smartphone. Proceedings of the International Conference on Information and Communication Technology Convergence.

[4] Abusara A., Hassan M.S., Ismail M.H. (2017). Reduced-complexity fingerprinting in WLAN-based indoor positioning. Telecommun. Syst..

[5] Xue W.X., Hua X.H., Li Q.Q., Yu K.G., Qiu W.N., Zhou B.D., Cheng K. (2018). A new weighted algorithm based on the uneven spatial resolution of RSSI for indoor localization. IEEE Access.

[6] Sayed A.H., Tarighat A., Khajehnouri N. (2005). Network-based wireless location: Challenges faced in developing techniques for accurate wireless location information. IEEE Signal Process. Mag..

[7] De Amorim R.C., Mirkin B. (2012). Minkowski metric, feature weighting and anomalous cluster initializing in K-means clustering. Pattern Recognit..

[8] Bezdek J.C., Ehrlich R., Full W. (1984). FCM: The fuzzy c-means clustering algorithm. Comput. Geosci..

[9] Goswami A. (2011). Wireless indoor localization using expectation-maximization on gaussian mixture models. Malar. J..

[10] Karegar P.A. (2018). Wireless fingerprinting indoor positioning using affinity propagation clustering methods. Wirel. Netw..

[11] Lee C., Lin T., Fang S., Chou Y. A novel clustering-based approach of indoor location fingerprinting. Proceedings of the Personal, Indoor and Mobile Radio Communications.

[12] Liu W., Fu X., Deng Z. (2016). Coordinate-based clustering method for indoor fingerprinting localization in dense cluttered environments. Sensors.

[13] Bi J., Wang Y., Li X., Qi H., Cao H., Xu S. (2018). An adaptive weighted KNN positioning method based on omnidirectional fingerprint database and twice affinity propagation clustering. Sensors.

[14] Fang X., Jiang Z., Lei N., Chen L. (2018). Optimal weighted K-nearest neighbour algorithm for wireless sensor network fingerprint localisation in noisy environment. IET Commun..

[15] Altintas B., Serif T. Improving RSS-based indoor positioning algorithm via K-means clustering. Proceedings of the Wireless Conference Sustainable Wireless Technologies European.

[16] Ma J., Li X., Tao X., Lu J. Cluster filtered KNN: A WLAN-based indoor positioning scheme. Proceedings of the International Symposium on World of Wireless, Mobile & Multimedia Networks.

[17] Shin B., Lee J.H., Lee T., Kim H.S. Enhanced weighted K-nearest neighbor algorithm for indoor Wi-Fi positioning systems. Proceedings of the International Conference on Computing Technology and Information Management.

[18] Lee I., Kwak M., Han D. (2016). A dynamic k-nearest neighbor method for WLAN-based positioning systems. Data Process. Better Bus. Educ..

[19] Nandakumar R., Chintalapudi K., Padmanabhan V.N. Centaur: Locating devices in an office environment. Proceedings of the ACM/IEEE International Conference on Mobile Computing and Networking.

[20] Conti A., Guerra M., Dardari D., Decarli N., Win M.Z. (2012). Network experimentation for cooperative localization. IEEE J. Sel. Areas Commun..

[21] Niu J., Lu B., Cheng L., Gu Y., Shu L. ZiLoc: Energy efficient WiFi fingerprint-based localization with low-power radio. Proceedings of the Wireless Communications and Networking Conference.

[22] Niu J., Wang B., Lei S., Duong T.Q., Chen Y. (2015). ZIL: An energy-efficient indoor localization system using ZigBee radio to detect WiFi fingerprints. IEEE J. Sel. Areas Commun..

[23] Gao Y., Niu J., Zhou R., Xing G. ZiFind: Exploiting cross-technology interference signatures for energy-efficient indoor localization. Proceedings of the International Conference on Computer Communications.

[24] Ma R., Guo Q., Hu C., Xue J. (2015). An improved WiFi indoor positioning algorithm by weighted fusion. Sensors.

[25] Liu X., Sheng Z., Zhao Q., Lin X. A novel approach for fingerprint positioning based on spatial diversity. Proceedings of the International Conference on Advanced Computer Theory & Engineering.

[26] Mirowski P., Ho T.K., Yi S., Macdonald M. SignalSLAM: Simultaneous localization and mapping with mixed WiFi, Bluetooth, LTE and magnetic signals. Proceedings of the International Conference on Indoor Positioning & Indoor Navigation.

[27] Ferris B., Fox D., Lawrence N.D. WiFi-SLAM using Gaussian process latent variable models. Proceedings of the International Joint Conference on Artifical Intelligence.

[28] Bi J., Wang Y., Li Z., Xu S., Zhou J., Sun M., Si M. (2019). Fast radio map construction by using adaptive path loss model interpolation in large-scale building. Sensors.

[29] Khalajmehrabadi A., Gatsis N., Akopian D. (2017). Structured group sparsity: A novel indoor WLAN localization, outlier detection, and radio map interpolation scheme. IEEE Trans. Veh. Technol..

[30] Song C., Wang J. (2017). WLAN fingerprint indoor positioning strategy based on implicit crowdsourcing and semi-supervised learning. ISPRS Int. J. Geo Inf..

[31] Jung S.H., Moon B.C., Han D. (2016). Unsupervised learning for crowdsourced indoor localization in wireless networks. IEEE Trans. Mob. Comput..

[32] Alshami I.H., Ahmad N.A., Sahibuddin S., Firdaus F. (2017). Adaptive indoor positioning model based on WLAN-fingerprinting for dynamic and multi-floor environments. Sensors.

[33] Xue W., Qiu W., Hua X., Yu K. (2017). Improved Wi-Fi RSSI measurement for indoor localization. IEEE Sens. J..

[34] He S., Lin W., Chan S.H. (2017). Indoor localization and automatic fingerprint update with altered AP signals. IEEE Trans. Mob. Comput..

[35] Zhuang Y., Syed Z., Li Y., El-Sheimy N. (2016). Evaluation of two WiFi positioning systems based on autonomous crowd sourcing on handheld devices for indoor navigation. IEEE Trans. Mob. Comput..

[36] Kohonen T. (2011). Learning Vector Quantization.

[37] Akl R.G., Tummala D., Li X. Indoor propagation modeling at 2.4 GHz for IEEE 802.11 networks. Proceedings of the IASTED International Multi Conference on Wireless and Optical Communications.

[38] De Souza R.S., Lins R.D. A new propagation model for 2.4 GHz wireless LAN. Proceedings of the Asia-Pacific Conference on Communications.

[39] Seidel S.Y., Rappaport T.S. (2002). 914 MHz path loss prediction models for indoor wireless communications in multifloored buildings. IEEE Trans. Antennas Propag..

[40] Davies D.L., Bouldin D.W. (1979). A cluster separation measure. IEEE Trans. Pattern Anal. Mach. Intell..

